# Supplementary material for: Splice-Junction-Based Mapping of Alternative Isoforms in the Human Proteome
Source: Cell Rep. Author manuscript; Available in PMC 2020 Jan 15. (PMC6961840; doi:10.1016/j.celrep.2019.11.026)
Supplement: 2 [file NIHMS1546469-supplement-2.zip › DF1/PXD006675/RightAtrium/RightAtrium_47_CAPN7_KVYYPGIFSMQLYFFK.pdf]

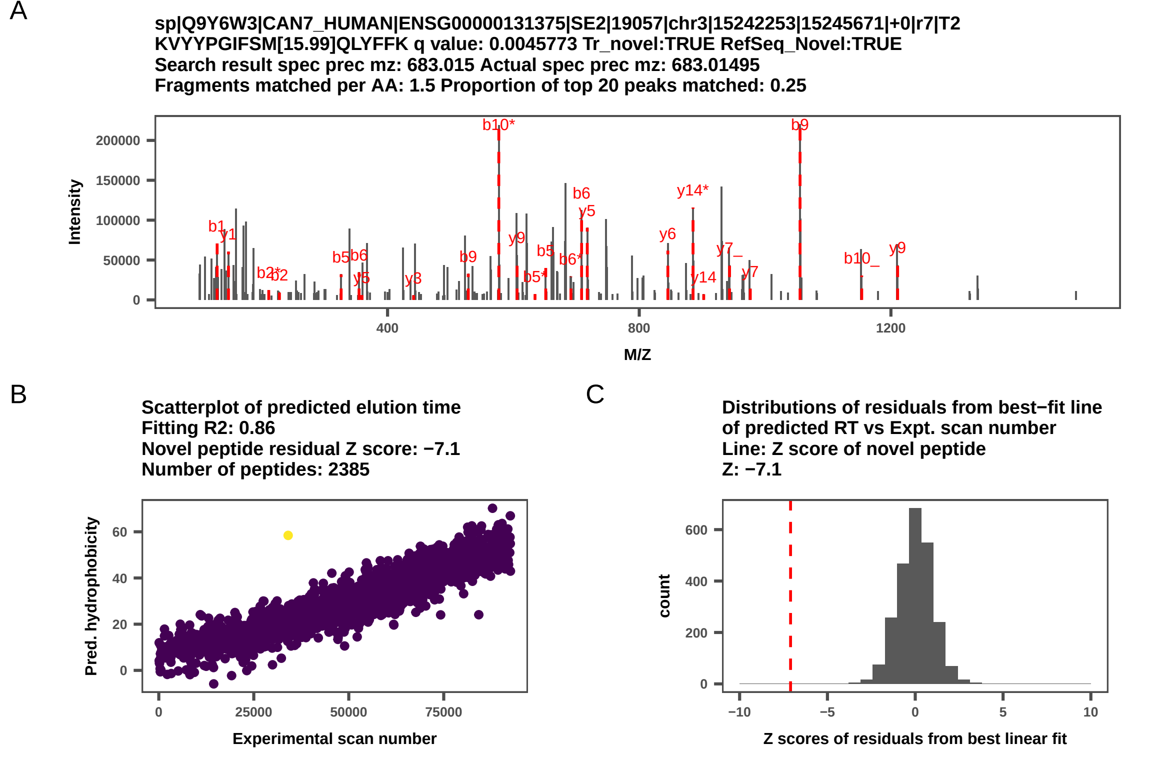
A
sp|Q9Y6W3|CAN7_HUMAN|ENSG00000131375|SE2|19057|chr3|15242253|15245671|+0|r7|T2
KVYYPGIFSM[15.99]QLYFFK q value: 0.0045773 Tr_novel:TRUE RefSeq_Novel:TRUE
Search result spec prec mz: 683.015 Actual spec prec mz: 683.01495
Fragments matched per AA: 1.5 Proportion of top 20 peaks matched: 0.25
Intensity
M/Z
B
Scatterplot of predicted elution time
Fitting R2: 0.86
Novel peptide residual Z score: −7.1
Number of peptides: 2385
Pred. hydrophobicity
Experimental scan number
C
Distributions of residuals from best−fit line
of predicted RT vs Expt. scan number
Line: Z score of novel peptide
Z: −7.1
count
Z scores of residuals from best linear fit
